# Microbial volatiles mediate bacterial evolutionary dynamics

**DOI:** 10.1038/s41396-023-01530-w

**Published:** 2023-10-17

**Authors:** Muhammad Syamsu Rizaludin, Paolina Garbeva, Mark Zwart, Jie Hu

**Affiliations:** 1 Department of Microbial Ecology, Netherlands Institute of Ecology (NIOO-KNAW), 6708 PB Wageningen, The Netherlands; 2 Department of Plant and Environmental Sciences, Faculty of Natural and Life Sciences, University of Copenhagen, 1871 Copenhagen, Denmark

**Keywords:** Microbial ecology, Evolution

Many microorganisms produce and respond to a range of structurally and functionally diverse volatiles. Microbial volatiles originate from a broad range of biosynthetic pathways and therefore display highly diverse structural and functional variations [1]. Volatile compounds are not only emitted into the gas phase but they can also be released in the water phase and play an important role in long-distance microbial interactions in both aquatic and terrestrial environments. As microbial volatiles can diffuse rapidly in both gas and water phases, they mediate swift chemical interactions and are the first compounds to reach a target organism [2]. The ability of microorganisms to release and respond to volatile compounds has been overlooked for a long time, but the knowledge gained in recent years shows that volatile-mediated interactions range from mutualism to competition.

Due to their hydrophobic and uncharged nature, volatiles are likely to penetrate cell membranes easily and can induce perturbations to target organisms, such as increased cell permeability. Thus, microbial volatiles can target different cell components including nucleoids, which will affect gene expressions. For example, volatiles were reported to influence motility, virulence, biofilm formation, secondary metabolite production, antibiotic resistance and growth of their interacting microorganisms [3].

Although many scientists now recognize that microbial volatiles could mediate eco-evolutionary dynamics [4], the recent article “Bacterial volatile organic compounds attenuate pathogen virulence via evolutionary trade-off” published in *The ISME Journal* [5] is among the first to use experimental evolution to explore the capacity for pathogen adaptation upon continuous exposure to microbial volatiles. The authors investigated how the plant-pathogenic bacteria *Ralstonia solanacearum* adapts to a blend of synthetic volatiles emulating those produced by biocontrol bacteria *Bacillus amyloliquefaciens* and how adaptation affects tradeoffs between tolerance to these volatiles vs. growth and virulence. They revealed increases in volatile tolerance of *R. solanacearum* that led to tradeoffs with growth in the absence of the volatile and virulence, resulting in reduced pathogenicity in planta. Hence, this work suggests that volatiles are important drivers of bacterial evolution: their use for biocontrol would not only immediately suppress the growth of a pathogen, but it would also result in selection for reduced virulence.

In comparison with other studies that usually test a single volatile compound, this study applied a mixture of 25 volatiles in the same ratio as they are produced by *B. amyloliquefaciens* after 3-day incubation in soil. The pathogen evolved in the presence and absence of a volatile blend for a period of 22 days to mimic the longer-term volatile exposure and give ample time for bacterial evolution. The authors then thoroughly investigated the phenotypic changes of the pathogen *R. solanacearum*, including volatile tolerance, antibiotic resistance, growth curve characteristics, and virulence traits in vitro and in planta. Genome sequencing of ancestral strains and evolved strains of *R. solanacearum* was conducted to explore the mutations and mechanisms underlying changes in volatile/antibiotic tolerance and virulence. The authors found highly convergent molecular evolution dominated by loss-of-function mutations in the *pilM* and *wecA* loci. These patterns explain the concurrent increase in antibiotic resistance observed and suggest it occurs because of reduced outer membrane permeability, a response induced by many stresses.

While it is practical to employ pure volatile compounds for dissecting their role (as individual or mixture) in the evolution of perceiving microorganisms, the involvement of volatile-emitting bacteria in the experimental setup should be considered in future studies. Hence, we not only consider a snapshot of volatiles detected at a certain period of microbial growth, but also consider the blend of volatiles produced by the microorganisms across their growth stages. In fact, the composition of microbial volatiles is never constant; it can vary depending on various parameters like the growth stage and physiological state of the producing microorganisms, oxygen availability, nutrient conditions, moisture, temperature, and pH in their growing environment [6, 7]. Monitoring of volatiles emitted by bacteria over time has shown that some compounds are produced in a transient manner.

What makes this new study particularly novel is the integration of chemical ecology and experimental evolution. The study employs a well-thought-out setup in terms of population size, replication and note three other salient features of the work. First, an interesting discrepancy in the results is the low bacterial titers during passaging but higher volatile tolerance for evolved clones [5], which the authors explain by invoking a tradeoff between growth and tolerance. Although growth in the presence of volatiles could increase without affecting the final yield, under the majority of plausible scenarios, increased fitness will result in higher yields. Here direct competition between ancestor and evolved clones under representative conditions—often the best choice for measuring fitness in experimental evolution [8, 9]—could lend more insight into the underlying mechanisms. Second, these results suggest epistatic interactions between beneficial mutations might be important here, given that neither individual loss-of-function mutation in *pilM* or *wecA* resulted in high volatile tolerance. Measuring the tolerance of a double mutant and determining when these mutations occurred during evolution could shed light on the importance of epistasis here. Third, the link between volatile tolerance and antibiotic resistance could be exploited for monitoring experimental populations at many time points using simple, high-throughput antibiotic-resistance assays. We hope this study will inspire many others to use evolutionary approaches to explore chemical ecology.

**Fig. 1:**
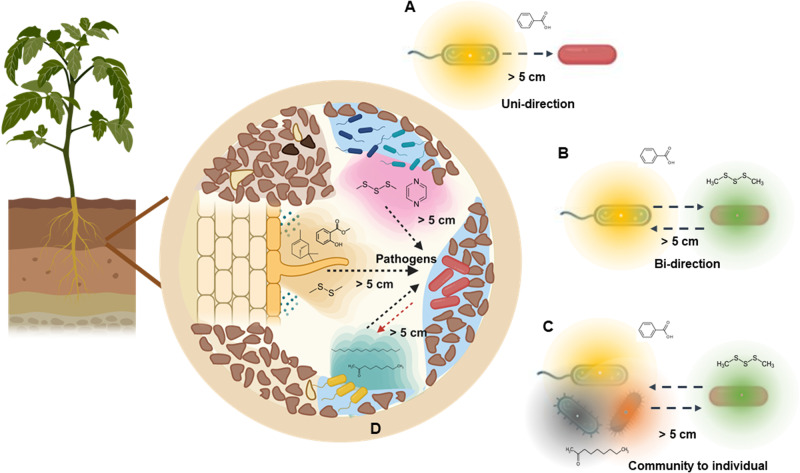
Interactions between beneficial microorganisms and one specific pathogen via volatile compounds under different scenarios. **A** Uni-direction interactions between individual beneficial microorganisms and one specific pathogen (microorganism in red color on the right) via volatiles (semi-transparent halo around microorganism on the left), representative of the work performed by Wang et al. [5]. **B** Bi-direction interactions between individual beneficial microorganisms and one specific pathogen via volatiles. **C** Interactions between beneficial microbial communities and one specific pathogen via volatiles. **D** More complex interactions between one specific pathogen and microbial communities in a relatively realistic soil-plant continuum via volatiles.

This recent study is focused on the uni-directional effect of volatiles produced by a single organism and the responses of the organisms perceiving them (Fig. 1A), without considering volatile-mediated dialog and bi-directional responses to one another (Fig. 1B). Bacteria can strongly influence each other, whether they belong to the same species or not, and this interaction can lead to the production of volatiles that they do not produce in isolation [10, 11]. Hence, microbial interactions can lead to the production of different volatiles from the ones produced in monocultures, adding another layer of complexity in investigating the evolutionary dynamic between the interacting species (Fig. 1C). Our understanding of the full implications of volatiles on the evolutionary trajectory of receiving microorganisms also remains limited as the majority of the data were obtained from in vitro experiments. In natural environments, however, such as soil and rhizosphere, microbial interactions are far more complex than single one-to-one interactions and involve more organisms which can significantly affect volatile-mediated interactions (Fig. 1D). Wang et al. have taken an important first step by considering a simple setup with controlled introduction of volatiles. This approach allows for higher repeatability of evolutionary patterns, given a single organism is adapting to a fixed environment. However, these questions call for studies on co-evolution and eventually the evolution of microbial communities, so that eco-evolutionary patterns can be studied under relevant conditions. Given the intricate and responsive nature of chemical communication, we cannot make meaningful predictions about the real-world evolutionary response of phytogenic bacteria to volatiles without considering community interactions.

In summary, the study of Wang et al. showed that microbial volatiles drive plant pathogen evolution by selecting volatile-tolerant mutants. The mutations associated with tolerance were also linked to reduced virulence in vitro and planta. Understanding the mechanisms by which microbial volatiles are involved in pathogen adaptation is crucial for harnessing beneficial microbes to sustainably control plant diseases in agroecosystems.
